# Analyzing fixed points of intracellular regulation networks with interrelated feedback topology

**DOI:** 10.1186/1752-0509-6-57

**Published:** 2012-06-06

**Authors:** Nicole Radde

**Affiliations:** 1 Institute for Systems Theory and Automatic Control University of Stuttgart Pfaffenwaldring 9, Germany

**Keywords:** Circuit-breaking algorithm, feedback circuit; fixed point analysis; fixed point bifurcation

## Abstract

**Background:**

Modeling the dynamics of intracellular regulation networks by systems of ordinary differential equations has become a standard method in systems biology, and it has been shown that the behavior of these networks is often tightly connected to the network topology. We have recently introduced the circuit-breaking algorithm, a method that uses the network topology to construct a one-dimensional circuit-characteristic of the system. It was shown that this characteristic can be used for an efficient calculation of the system’s fixed points.

**Results:**

Here we extend previous work and show several connections between the circuit-characteristic and the stability of fixed points. In particular, we derive a sufficient condition on the characteristic for a fixed point to be unstable for certain graph structures and demonstrate that the characteristic does not contain the information to decide whether a fixed point is asymptotically stable. All statements are illustrated on biological network models.

**Conclusions:**

Single feedback circuits and their role for complex dynamic behavior of biological networks have extensively been investigated, but a transfer of most of these concepts to more complex topologies is difficult. In this context, our algorithm is a powerful new approach for the analysis of regulation networks that goes beyond single isolated feedback circuits.

## Background

Describing the dynamic behavior of molecular interactions in a cell or cell compartment by chemical reaction kinetics has become a standard approach in systems biology for metabolic pathways as well as for regulatory networks. Since qualitative knowledge about these interactions is often available from experiments, literature or databases, which can be represented as network graphs, several different graph-based approaches have been developed to analyze the behavior of the networks. These methods operate solely on the graphs without detailed knowledge of the kinetic rates. They show for example that certain subnetwork structures are necessary to generate complex behavior such as oscillations, hysteresis or multistationarity. Thus, such behavior can be excluded for relatively small and simple networks that lack these subnetworks. So far, most of these approaches have the following limitations for practical use: First, they only allow to make statements for relatively simple graph topologies, and second, they are often restricted to very specific model classes such as metabolic networks of the form $\dot{x}=\mathrm{Sv}(x)$ with stoichiometric matrix *S* and (often polynomial) flux vector *v*(*x*) [1] or regulatory networks whose Jacobian matrices have constant signs on the off-diagonal elements [2-5]. Similar analysis methods that work for more complex graph topologies or more general network model classes are rare. On the other hand, it has been shown in various contexts that interrelated feedback structures contribute to the robustness of intracellular regulation processes [6-13]. In most studies this is demonstrated by analyzing a specific model via simulations with varying parameter values, for example via Monte Carlo simulations. Although the conclusions from these studies are very helpful and valuable, it is not clear to which extend they can be generalized to other network models. These results, which show the importance of feedback in regulation processes, provide a further basis for the need of new methods that can deal with interrelated feedback in dynamic network models in a more general way. We expect that the more complex the graph topologies, the more does the system’s behavior depend on the kinetic rate laws, and less can be concluded from the structure alone. Thus, these new methods can probably not be completely independent of equations and parameters.

A new approach in this direction has been introduced in our previous work [14] for a general class of regulatory network models. We introduced the *circuit-breaking algorithm* (CBA), a method which operates on the graph topology to construct a one-dimensional characteristic that inherits important information about the behavior of the system. In particular, we demonstrated that the zeros of this characteristic are related to the system’s fixed points.

In this paper we extend this work and show that the characteristic contains information about stability of the fixed points and can furthermore be used to detect bifurcation point candidates. The paper is structured as follows: We give a brief overview over our network model class and the circuit-breaking algorithm and show how it works on a network for cellular differentiation of hematopoietic stem cells [15]. Based on these results, we investigate relations between the stability of fixed points and the slope of the circuit-characteristic that is constructed by the CBA. It is shown that a negative slope at a zero of the characteristic does generally not contain any information about the stability of the respective fixed point, while a positive slope implies that the fixed point is unstable, at least for certain graph topologies. We demonstrate results on biological network models for tryptophan regulation in *Escherichia coli*[11] and the repressilator model [16].

## Results and Discussion

### The circuit-breaking algorithm

Here we introduce the *regulatory network model* class and summarize the concept of the CBA. For details we refer to [14]. Since the formal description of the algorithm is very technical and needs a lot of indices, we will thereafter directly show how it works on a concrete network example, from which we hope that it makes the general concept easier understandable.

We consider regulatory networks models that are described by a system of first order ordinary differential equations

(1) $$\dot{x}=f(x),\;x\in R^{n},f\in C^{1}$$

with underlying *interaction graph* (I-graph) *G*(*V *,*E*) that illustrates the dependence structure of the variables, i.e.

(2) $$e_{j\to i}\in E\;\Leftrightarrow \;\exists \;x\in R^{n}\;\text{such that}\;\frac{\partial {\dot{x}}_{i}(x)}{\partial x_{j}}\neq 0$$

and

(3) $$e_{i\to i}\in E\;\Leftrightarrow \;\exists \;x\in R^{n}\;\text{such that}\;\frac{\partial {\dot{x}}_{i}(x)}{\partial x_{i}}>0.$$

Trajectories of the system should be bounded, a biologically plausible assumption which also implies that the system has at least one fixed point. It is sometimes useful for the analysis to introduce sign-labels of edges in the I-graph if the corresponding partial derivative is monotone, which means that the influence of a component on another one is purely activating or purely inhibiting regardless of the state of the system. Contrary to many other methods, the CBA does not rely on this monotonicity assumption.

**Figure 1:**
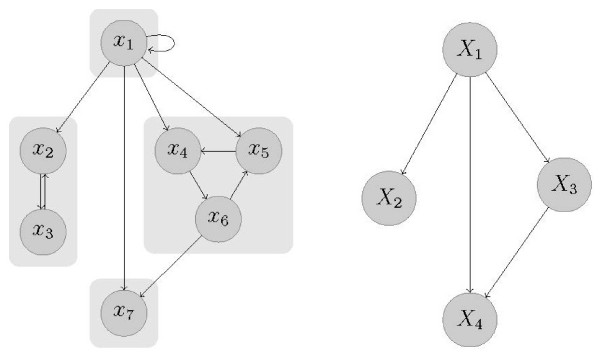
**Division of vertices into strongly connected components.** An example of a graph *G*(*V *,*E*) and it’s partitioning into strongly connected components (*left*). Within a strongly connected component, each pair of vertices is connected via a path. If each SCC is contracted to a single vertex, the resulting graph is circuit-free (*right*) (if it contained circuits, the SCCs are not maximal, since all SCCs in the circuit could be merged to a larger SCC) and thus has a hierarchical order.

Given a regulatory network model, i.e. a differential equation system $\dot{x}=f(x)$ and the I-graph topology *G*(*V *,*E*), the CBA consists of the following steps:

1. *Find strongly connected components of G(V,E):*The first step of the CBA is a partitioning of the vertex set *V* into strongly connected components (SCC), i.e. the maximal strongly connected subgraphs, which we denote by *G*^*k*^(*V*^*k*^*E*^*k*^), *k*=1,…,*K*. The new graph *G*^*SCC*^(*V*^*SCC*^*E*^*SCC*^), which is obtained by shrinking all vertices of a SCC to one single vertex and drawing an edge $e_{i\to j}^{\mathrm{SCC}}$ between two vertices $v_{i}^{\mathrm{SCC}}$ and $v_{j}^{\mathrm{SCC}}$ of this graph when there exist vertices $v_{i}\in V^{i}$ and $v_{j}\in V^{j}$ that are connected with an edge $e_{i\to j}$ in the original graph *G*(*VE*), has a hierarchical topology without any circuits. This fact is illustrated in Figure 1.We numerate the SCCs according to this hierarchical order in the network. Fixed point coordinates of the system can iteratively be calculated for each SCC, starting with the SCC on top and following the hierarchical structure downwards. In this scheme the fixed point coordinates of the SCCs upstream serve as constant input *u* for subsequent SCCs. An example for this concept of iterative fixed point calculation for SCCs is given in [14]. We denote these sets of fixed points of SCC *k* with input *u* by ${}_{u}F^{k}$. For the sake of simplicity we skip the index *u* in the following, but bear in mind that the fixed point set *F*^*k*^has to be calculated for each input *u*.

2. *Construct characteristics for each SCC in dependence of input u and calculate the fixed points from it’s zeros:* The core of the CBA is the construction of a one-dimensional characteristic $c^{k}(\kappa_{1}^{k})$ for a SCC *G*^*k*^(*V*^*k*^,*E*^*k*^) for each input *u*. This is done in the following way: 

(a) Find the set *C* of all elementary circuits and list them as set of vertex subsets

(b) Find a minimal circuit-covering vertex set $\tilde{V}$ such that at least one element of each subset in *C* is contained in $\tilde{V}$ and set $m=|\tilde{V}|$. Collect the rest of the vertices in the set $\hat{V}$. Relabel vertices such that $\tilde{V}=\{v_{1},\ldots ,v_{m}\}$ and $\hat{V}=\{v_{m+1},\ldots ,v_{\left|V^{k}\right|}\}$.

(c) Break all circuits by removing all edges that point to vertices of $\tilde{V}$. Mathematically, this is done by setting these variables to fixed input values *κ*=(*κ*_1_,…,*κ*_*m*_), i.e. *x*_*i*_=*κ*_*i*_. The result is an acyclic or hierarchical graph topology.

(d) The fixed point coordinates of variables in $\hat{V}$, denoted by $F(\kappa )={\left\{{\bar{x}}_{p}(\kappa )\right\}}_{p=m+1,\ldots ,|V^{k}|}$, can be calculated in dependence of these inputs *κ*.

(e) The circuits are iteratively closed by releasing the vertices in $\tilde{V}$ one after another, starting backwards with *v*_*m*_. This translates into shifting the respective vertex *v*_*i*_ from the set $\tilde{V}$ to $\hat{V}$, reducing the vector *κ*by the same element, and solving the implicit equation of the form

(4) $$f_{i}(x_{i},\kappa ,F(\kappa ))=0$$

for *x*_*i*_ to get the set ${\bar{x}}_{i}(\kappa )$ of fixed point coordinates of the variable *x*_*i*_ in dependence of the vector *κ*. The set *F*(*κ*) has to be updated accordingly. Equation (4) has to be solved numerically. For *i*=2,…,*m*we denote the expression on the left hand side of equation (4) *partial circuit-characteristic*. The number of input variables of these characteristics is reduced by one in each step, since *κ*is reduced by one element in each step. Thus, when releasing the last vertex *v*_1_ in $\tilde{V}$, $f_{1}(x_{1}=\kappa_{1},F(x_{1}=\kappa_{1})):R\to R$ is a one-dimensional characteristic that is called the *circuit-characteristicc*(*κ*_1_) of the respective SCC. It’s zeros correspond to the fixed point coordinates of variable *x*_1_, denoted by $\left\{{\bar{x}}_{1}\right\}$. If we leave the current SCC and go to the next one, we refer to this characteristic as $c^{k}(\kappa_{1}^{k})$.

(f) The corresponding fixed point coordinates of the other variables can be calculated iteratively by inserting the values of the set $\left\{{\bar{x}}_{1}\right\}$ into the partial circuit-characteristics in reverse order. These coordinates are then collected in the set *F* of fixed point coordinates of the SCC k. If we leave the SCC *k*, we refer to this set as *F*^*k*^.

**Figure 2:**
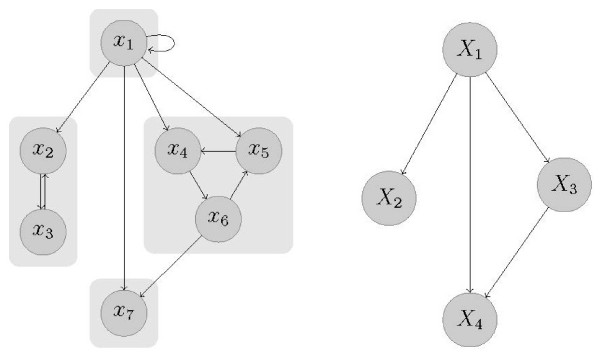
**Flow chart of the Circuit-Breaking Algorithm.** Flow chart of the Circuit-Breaking Algorithm. Blue boxes indicate that these calculations are done within a SCC of the graph, yellow boxes describe the iterative closing of the circuits within this SCC by releasing vertices in the set ${\tilde{V}}^{k}$ one after another. The green boxes refer to actions on the full graph *G*(*V *,*E*).

The structure of the CBA is illustrated in Figure 2 with a flow chart.

### Application of the CBA to a model for hematopoietic stem cell differentiation

To motivate the subsequent investigations on the characteristics of regulatory network models and it’s relation to fixed points and their stability, we consider a network model for the cellular differentiation of hematopoietic stem cells described in [15]:

(5) $$\begin{array}{lll} {\dot{x}}_{1}\;=\; & e_{N}-x_{1} & \;=\;r_{1}-x_{1}\\ {\dot{x}}_{2}\;=\; & \frac{5x_{1}}{1+x_{1}}\frac{1}{1+x_{3}^{4}}-x_{2} & \;=\;r_{2}(x_{1},x_{3})-x_{2}\\ {\dot{x}}_{3}\;=\; & \frac{5x_{4}}{1+x_{4}}\frac{1}{1+x_{2}^{4}}-x_{3} & \;=\;r_{3}(x_{2},x_{4})-x_{3}\\ {\dot{x}}_{4}\;=\; & \frac{e_{M}}{1+x_{2}^{4}}-x_{4} & \;=\;r_{4}(x_{2})-x_{4}\\ {\dot{x}}_{5}\;=\; & \left(\frac{x_{1}x_{4}}{1+x_{1}x_{4}}\;+\;\frac{4x_{3}}{1+x_{3}}\right)\frac{1}{1+x_{2}^{4}}\;-\;x_{5} & \;=\;r_{5}(x_{1},x_{2},x_{3},x_{4})\;-\;x_{5}\\ {\dot{x}}_{6}\;=\; & \left(\frac{x_{1}x_{4}}{1+x_{1}x_{4}}\;+\;\frac{4x_{2}}{1+x_{2}}\right)\frac{1}{1+x_{3}^{4}}\;-\;x_{6} & \;=\;r_{6}(x_{1},x_{2},x_{3},x_{4})\;-\;x_{6} \end{array}$$

This model describes the interplay between the two lineage-specific counter-acting suppressors Gfi-1 (*x*_2_) and Egr(1,2) (*x*_3_) during cellular differentiation for the neutrophil and macrophage cell fate choices, respectively. These are activated by their transcription factors C/EBP*α*(*x*_1_) and PU.1 (*x*_4_), respectively. Together, they regulate the expression of lineage-specific downstream genes, which are not further specified in the model and denoted by Mac (*x*_5_) and Neut (*x*_6_). The model was build based on chemical reaction kinetics that describe interaction of the molecular species. The cellular state is assumed to be directly correlated to the fixed point concentrations of the transcription factors, as described further below. Furthermore, the model was non-dimensionalized after some simplifications by rescaling time and protein concentrations. The two parameters that are left, *e*_*N*_ and *e*_*M*_, are the rescaled synthesis rate of the transcription factor C/EBP*α*, which is not regulated in the model, and the maximal rescaled synthesis rate of the transcription factor PU.1.

**Figure 3:**
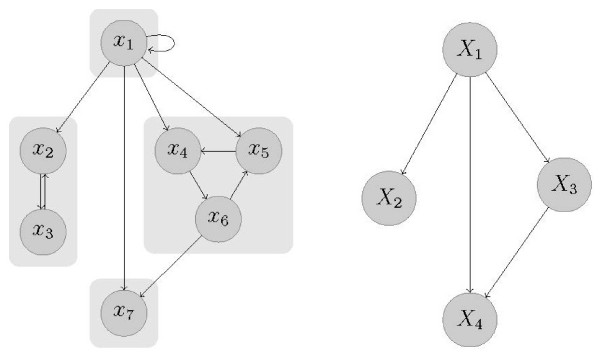
**Bifurcation diagrams of a model for cellular differentiation of hematopoietic stem cells.** Bifurcation diagrams of the hematopoietic stem cell differentiation network (system (5)) described in [15] with bifurcation parameter *μ*=*e*_*M*_. Has been created with Additional file 1.

Figure 3 shows the bifurcation diagram of all six variables with bifurcation parameter *μ*=*e*_*M*_ and condition *e*_*N*_=*e*_*M*_ that was created using xpppaut. For *e*_*M*_=0 the system has a globally stable fixed point at *x*=0. The system undergoes a saddle-node bifurcation at $e_{M}^{*}\approx 0.3221$. It has a globally stable fixed point for $e_{M}<e_{M}^{*}$ and two stable fixed points divided by an intermediate unstable one for $e_{M}>e_{M}^{*}$. It can also be seen that the stable fixed point branch that exists for all *e*_*M*_represents the neutrophil state, since the fixed point coordinates of the neutrophil specific proteins (*x*_1_,*x*_2_,*x*_6_) increase monotonically along this branch. The macrophage state is represented by the stable fixed point branch that appears at $e_{M}^{*}$.

**Figure 4:**
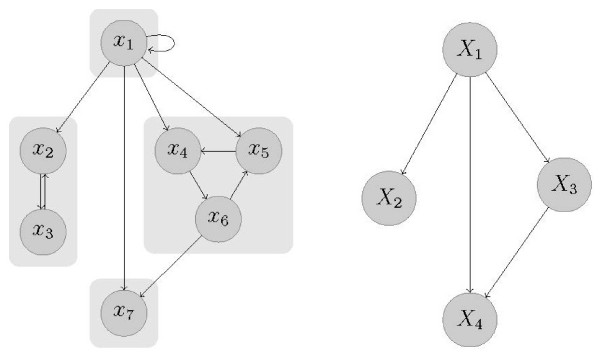
**I-graph of a model for cellular differentiation of hematopoietic stem cells.** I-graph *G*(*V *,*E*) of system (5). It consists of four SCCs, as indicated with the grey boxes, and *C*^2^has two interrelated positive circuits.

Now we use the CBA to construct the characteristic of this system and compare this with the information of the bifurcation diagram. As can be seen in Figure 4, the I-graph of system (5) consists of four strongly connected components given by $V^{1}=\{x_{1}\},V^{2}=\{x_{2},x_{3},x_{4}\},V^{3}=\{x_{5}\}$ and $V^{4}=\{x_{6}\}$ with circuit sets $C^{1}=C^{3}=C^{4}=\emptyset ,C^{2}=\left\{\left\{x_{2},x_{4},x_{3}\},\{x_{2},x_{3}\right\}\right\}$, and minimal circuit-covering vertex sets ${\tilde{V}}^{1}={\tilde{V}}^{3}={\tilde{V}}^{4}=\emptyset$ and ${\tilde{V}}^{2}=\{x_{2}\}$.

We start with *G*^1^(*V*^1^,*E*^1^), which does not contain any circuits. Thus, we just have to solve ${\dot{x}}_{1}=0$ in system (5), which leads to the set $F^{1}={\bar{x}}_{1}^{1}=\{r_{1}\}$ of fixed points of *G*^1^. The fixed point set ${}_{u=r_{1}}F^{2}=\{{\bar{x}}_{2}^{2}(x_{1}=r_{1}),{\bar{x}}_{3}^{2}(x_{1}=r_{1}),{\bar{x}}_{4}^{2}(x_{1}=r_{1})\}$ of *G*^2^(*V*^2^,*E*^2^) is calculated by breaking the two circuits at *x*_2_, i.e. setting $x_{2}=:\kappa_{1}^{2}$. Inserting ${\bar{x}}_{4}(\kappa_{1}^{2})=r_{4}(\kappa_{1}^{2})$ and ${\bar{x}}_{3}(\kappa_{1}^{2})=r_{3}(\kappa_{1}^{2},r_{4}(\kappa_{1}^{2}))$ into ${\dot{x}}_{2}$ leads to the circuit-characteristic

(6) $$u=r_{1}c^{2}(\kappa_{1}^{2})=r_{2}\left(r_{1},r_{3}\left(\kappa_{1}^{2},r_{4}(\kappa_{1}^{2})\right)\right)-\kappa_{1}^{2},$$

which can, by inserting the respective terms for the synthesis rates *r*, be rewritten as

(7) $$c^{2}(\kappa_{1}^{2})=\frac{5r_{1}}{1+r_{1}}\frac{1}{1+{\bar{x}}_{3}(\kappa_{1}^{2})}-\kappa_{1}^{2}$$

with

(8) $$\begin{array}{cc} {\bar{x}}_{3}(\kappa_{1}^{2}) & =\frac{5{\bar{x}}_{4}(\kappa_{1}^{2})}{1+{\bar{x}}_{4}(\kappa_{1}^{2})}\frac{1}{1+{\left(\kappa_{1}^{2}\right)}^{4}}\;\text{and}\\ {\bar{x}}_{4}(\kappa_{1}^{2}) & =\frac{e_{M}}{1+{\left(\kappa_{1}^{2}\right)}^{4}}. \end{array}$$

**Figure 5:**
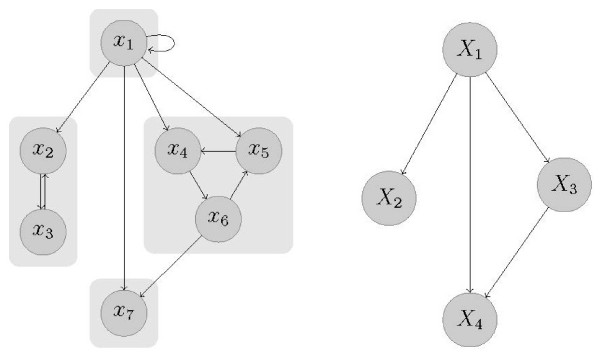
**Circuit characteristics of a model for cellular differentiation of hematopoietic stem cells.** From top to bottom: Sets ${\bar{x}}_{3}(\kappa_{1}^{2})$, ${\bar{x}}_{4}(\kappa_{1}^{2})$, circuit-characteristic $c^{2}(\kappa_{1}^{2})$, ${\bar{x}}_{5}(\kappa_{1}^{2})$ and ${\bar{x}}_{6}(\kappa_{1}^{2})$ for values *e*_*M*_=0.2 (*left column*), $e_{M}^{*}=0.3221$ (*center column*) and *e*_*M*_=0.5 (*right column*) of system (5). The fixed points that correspond to the zeros of the characteristic are also indicated. Has been created by Additional file 2.

This characteristic is shown in Figure 5 (*center row*), along with the sets ${\bar{x}}_{i}(\kappa_{1}^{2})$, *i*=3,4,5,6, for parameter values *e*_*M*_={0.2,0.3221,0.5} (*left, center, right row*, respectively).

The following properties of the system can be identified from these figures: 

1. The fixed point coordinates of all variables *x*_3_,*x*_4_,*x*_5_and *x*_6_behave monotonically with the input *κ*_2_, which represents the neutrophil state. The macrophage specific proteins *x*_3_,*x*_4_and *x*_5_decrease with increasing $\kappa_{1}^{2}$, *x*_6_increases.

2. Looking at the characteristics (*center row*) for different values *e*_*M*_, it is monotonically decreasing for $e_{M}<e_{M}^{*}$ (*left*), and thus has a single zero, which corresponds to the single fixed point branch for $e_{M}<e_{M}^{*}$. For the value *e*_*M*_=0.2, which is chosen here, we get the fixed point $\bar{x}(\mu =0.2)$={0.2,0.81,0.43,0.14,0.86,1.75}, as indicated in the graphs. This state represents an intermediate non-differentiated progenitor cell state. The saddle-node bifurcation is represented by the second zero of the characteristic that appears at $e_{M}=e_{M}^{*}$ (*center column*). The respective fixed point set is ${\bar{x}}^{1}(\mu =0.3221)=\{0.3221,0.51,1.09,0.30,2.04,$0.59} and ${\bar{x}}^{2}(\mu =0.3221)=\{0.3221,1.21,0.15,$0.10,0.17,2.22}.Finally, the characteristic has three zeros for $e_{M}>e_{M}^{*}$ (*right column*) and thus the system has three fixed points in this range. For the chosen value *e*_*M*_=0.5 we can read off the fixed point set ${\bar{x}}^{1}(\mu =0.5)=\{0.5,0.20,1.67,0.5,2.7,0.03\}$, ${\bar{x}}^{2}(\mu =0.5)=\{0.5,0.75,1.05,0.38,1.68,0.84\}$ and ${\bar{x}}^{3}(\mu =0.5)=\{0.5,1.66,0.03,0.06,0.02,2.52\}$. Here, ${\bar{x}}^{1}$ represents the macrophage state, where Egr and PU.1 are highly expressed, and C/EBP*α*is low, ${\bar{x}}^{3}$ stands for the neutrophil state in which C/EBP*α*is dominant, and ${\bar{x}}^{2}$ is an unstable intermediate state that separates the two basins of attraction.

Seeking for further parallels between the bifurcation diagram (Figure 3) and the characteristics in Figure 5, the question arises if the characteristic also contains information about bifurcations and stability of the fixed points. Clearly, the parameters for which the characteristic touches the x-axis without intersection are bifurcation value candidates. Furthermore, looking at this example, a self-evident guess would be to assume that stability can be determined in the same way as for one-dimensional vector fields: The fixed points are stable if the slope of the characteristic at the corresponding zero *κ*^∗^ is negative, i.e. $\mathrm{dc}(\kappa )/\mathrm{d\kappa}{\left|\right)}_{\kappa =\kappa^{*}}<0$, and it is unstable if the slope is positive, i.e. $\mathrm{dc}(\kappa )/\mathrm{d\kappa}{\left|\right)}_{\kappa =\kappa^{*}}>0$. We will further investigate these assumptions in the following subsections. In order to do so, we consider in the following strongly connected I-graphs, which allows to neglect the indices *u* and *k*, such that indexing can be simplified. The results are, however, easily transferable to arbitrary graphs, since construction of the characteristic is done separately for each strongly connected component. We will continue by denoting the characteristic simply with *c*(*κ*_1_), where $\kappa_{1}\in R$ is the value of the variable *x*_1_, the one which is released lastly. We first prove the following proposition, which relates the slope of the characteristic to the determinant of the Jacobian matrix *J*_*f*_(*x*) of the system:

#### Proposition 1

(9) $$\frac{\mathrm{dc}(\kappa_{1})}{d\kappa_{1}}\;\;=\;\;\frac{df_{1}(x)}{dx_{1}}{\left|\right)}_{\left(x_{1},F(x_{1})\right)}$$

(10) $$\;=\;\det\;J_{f}(x){\left|\right)}_{\left(x_{1},F(x_{1})\right)}\cdot \det^{-1}J_{f}^{V\setminus \{v_{1}\}}(x){\left|\right)}_{F(x_{1})}$$

with *F*(*x*_1_) denoting the fixed point coordinates of variables *x*_2_,…,*x*_|*V*|_in dependence of *x*_1_, and $J_{f}^{V\setminus \{v_{1}\}}(x)$ is the Jacobian matrix of the subnetwork spanned by the vertices *V*∖{*v*_1_}.

The proof is given in the Methods section. Note that Proposition 1 holds for all inputs *κ*_1_, but we are here especially interested in the zeros of the characteristic, i.e. the set of $\kappa_{1}^{*}$ with $c(\kappa_{1}^{*})=0$, and we will in the following subsection sometimes denote the corresponding fixed point with $\bar{x}$, if appropriate.

### Instability of fixed points

From Proposition 1 it follows that a positive slope $\frac{\mathrm{dc}(\kappa_{1})}{d\kappa_{1}}{\left|\right)}_{\kappa_{1}^{*}}>0$ implies that $\det\;J_{f}(\bar{x}){\left|\right)}_{\left({\bar{x}}_{1},F({\bar{x}}_{1}))\right)}$ and $\det\;\overset{V\setminus \{v_{1}\}}{\underset{f}{J}}(\bar{x}){\left|\right)}_{F({\bar{x}}_{1})}$ have the same signs. According to the Hartman-Grobman theorem (see e. g. [17]), a fixed point $\bar{x}$ is unstable if $J_{f}(\bar{x})$ has at least one eigenvalue with positive real part. Unfortunately, we are not aware of a relation between the determinant of *J*_*f*_(*x*) and it’s minors that can be used to show the following: If $\det\;J_{f}(\bar{x})$ and $\det\;\overset{V\setminus \{v_{1}\}}{\underset{f}{J}}(\bar{x})$ have the same signs, this implies the existence of an eigenvalue with positive real part and hence implies instability of $\bar{x}$. Thus we will concentrate on certain graph structures which we call *leading vertex graphs* (LVG). LVGs are strongly connected components with minimal circuit covering vertex set $\tilde{V}$ that consists of one single element *v*_1_. In other words, *G*(*V E*) has a vertex that is contained in all elementary circuits, and hence the characteristic *c*(*κ*_1_) can be constructed in a single circuit-closing step. The I-graph of the hematopoietic stem cell differentiation network consists of SCCs that are all LVGs, while the two networks considered in the proof of proposition (1) do not belong to this class, because two circuit-closing steps were necessary in each of these cases. For LVGs we can show that a positive slope of the characteristic at a zero implies instability of the respective fixed point. The proof is given in the Methods section.

### Stability of fixed points

**Figure 6:**
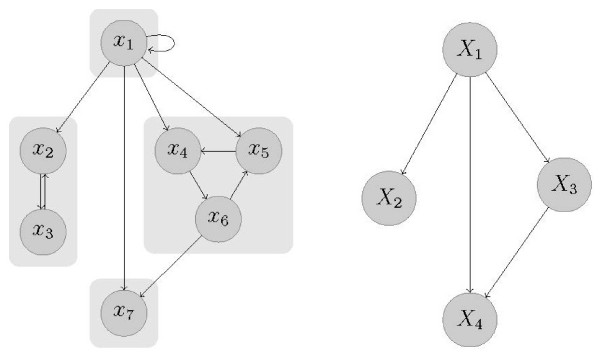
**I-graph of the repressilator model.** I-graph of the repressilator model (11) described in [16].

In contrast to a positive slope, a negative slope of the circuit-characteristic at a fixed point coordinate *κ*^∗^ alone does not contain information about the stability of the respective fixed point. We demonstrate this with two examples. The first one consists of a single negative feedback circuit, the repressilator model described in [16]. This is a synthetic transcriptional network of the three repressor proteins lacI, tetR and cI and their corresponding promoters, which was constructed to create periodic expression in *Escherichia coli*:

(11) $$\begin{array}{lll} {\dot{m}}_{i} & =\frac{\alpha }{1+p_{j}^{n}}+\alpha_{0}-m_{i}=:r(p_{j})-m_{i}\; & \;\\ {\dot{p}}_{i} & =\beta (m_{i}-p_{i}),\; \end{array}$$

with *i*={lacI,tetR,cI}, *j*={cI,lacI,tetR}, and *m*_*i*_ and *p*_*i*_ are mRNA and protein concentrations, respectively. The system has a trapping region, that is, a positively invariant region in the state space that is eventually reached by all trajectories, which guarantees the existence of at least one fixed point. Bounds are given by $m_{i}^{\min}=\alpha_{0}$$m_{i}^{\max}=\alpha +\alpha_{0}$ and $p_{i}^{\min/\max}=m_{i}^{\min/\max}$*i*=1,2,3. The I-graph (Figure 6) is strongly connected, the circuit set *C* consists of one subset that contains all six nodes, *C*={{*m*_*i*_*p*_*i*_}_*i*=1,2,3_}, and hence the set $\tilde{V}$ has one single element and the graph is a LVG.

Note that because of the symmetry of the model, the circuit-characteristic is independent of the choice of $\tilde{V}$ here. It is given by

(12) $$c(\kappa_{1})=r({\bar{p}}_{3}({\bar{m}}_{3}({\bar{p}}_{2}({\bar{m}}_{2}({\bar{p}}_{1}(\kappa_{1}))))))-\kappa_{1},$$

which can be simplified to

(13) $$c(\kappa_{1})=r(r(r(\kappa_{1})))-\kappa_{1},$$

where we have used ${\bar{p}}_{i}({\bar{m}}_{i})={\bar{m}}_{i}$, ${\bar{m}}_{i}({\bar{p}}_{j})=r({\bar{p}}_{j})$ and ${\bar{p}}_{1}={\bar{p}}_{2}={\bar{p}}_{3}$. Equation (13) is strictly decreasing, and, importantly, independent of the parameter *β*.

**Figure 7:**
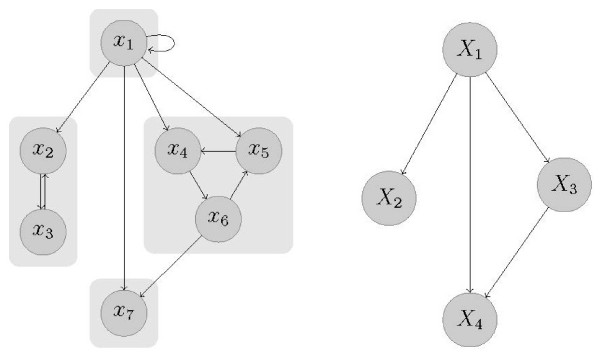
**Eigenvalues of the repressilator model.** Real (green) and imaginary (red) parts of the eigenvalues *λ*(*β*) of $J_{f}(\bar{x})$ of the repressilator model (11) with parameters *α*=290, *n*=2, _*α*0_=10 and ${\bar{m}}_{i}={\bar{p}}_{i}=12$. The figure was created by calculating the characteristic polynomial *χ*(*λ*,*β*) of $J_{f}(\bar{x})$, which is given here as $\chi (\lambda ,\beta )={\left(1+\lambda \right)}^{3}{\left(\beta +\lambda \right)}^{3}-\beta^{3}r^{\prime \prime }{\left(\bar{p}\right)}^{3}$ with $r^{\prime \prime }(\bar{x})=\frac{-2\alpha \bar{p}}{{\left(1+{\bar{p}}^{2}\right)}^{2}}\approx 0.33$, and using a Newton gradient search with several random starting points to find the eigenvalues *λ*with accuracy $\left|\det(J_{f}(x)-\lambda (\beta )I)\right|$<10^−4^. Has been created with Additional files 3 and 4.

On the contrary, the eigenvalues of the Jacobian matrix of the system and hence the stability of the fixed point are not (see also the stability diagram in Figure 1b in [16]). This dependence is illustrated in Figure 7, where the real and imaginary parts of the eigenvalues *λ*(*β*) of the Jacobian matrix $J_{f}(\bar{x})$ are plotted as functions of *β* for parameter values *α*=290, *n*=2 and *α*_0_=10. For these parameter values the system has a fixed point ${\bar{m}}_{i}={\bar{p}}_{i}=12$ for all *i*=1,2,3 (that is independent of *β*). It can be seen that there exist solutions with positive real part for small values of *β*, and hence the fixed point is unstable in this range. It becomes stable through a Hopf bifurcation for increasing values of *β*. Thus we have shown that $J_{f}(\bar{x})$ and in particular the stability of $\bar{x}$ depend on *β*, while *c*(*κ*_1_) does not. From this example we conclude that our assumption is not true for zeros of the characteristic with negative slope. The corresponding fixed point of the system can generally be stable or unstable. In the Methods section proposition 1 is verified for this example.

As a further example we consider the tryptophan regulation network in *Escherichia coli* described in [11], which can be written as

(14) $$\;\begin{array}{lll} {\dot{x}}_{1}\;= & k_{1}O_{t}C(x_{4},\;t_{1},m_{1})\;-\;(\gamma_{1}\;+\;\mu )x_{1} & \;=\;r_{1}(x)\;-\;(\gamma_{1}\;+\;\mu )x_{1}\\ {\dot{x}}_{2}\;= & k_{2}x_{1}C(x_{4},\;t_{2},\;m_{2})\;-\;(\gamma_{2}\;+\;\mu )x_{2} & =r_{2}(x)\;-\;(\gamma_{2}\;+\;\mu )x_{2}\\ {\dot{x}}_{3}= & k_{3}x_{2}-\mu x_{3} & =r_{3}(x)-\mu x_{3}\\ {\dot{x}}_{4}= & k_{4}C(x_{4},t_{3},m_{3})x_{3}-g\frac{x_{4}}{x_{4}+K_{g}}-\mu x_{4} & =r_{4}(x)-\mu x_{4}, \end{array}$$

where the state vector *x* corresponds to the free operator sites (*O*_*R*_), mRNA (M), enzyme (E) and tryptophan (T) concentrations. *C*(*xKm*) are sigmoidally decreasing functions,

(15) $$C(x,K,m)=\frac{K^{m}}{K^{m}+x^{m}}.$$

This model describes the regulation of the tryptophan concentration via different mechanisms, i.e. genetic regulation via binding of tryptophan to it’s operator site, described by *C*(*x*_4_*t*_1_*m*_1_), mRNA attenuation (*C*(*x*_4_*t*_2_*m*_2_)) and enzyme inhibition (*C*(*x*_4_*t*_3__*m*3_)). The parameters *k*_1_*k*_2_*k*_3_and *k*_4_represent kinetic rate constants for synthesis of free operator, mRNA transcription, translation and tryptophan synthesis, respcetively, *K* are half-saturation constants for the inhibition processes, *O*_*t*_ denotes the total operator site concentration, and *γ*and *μ*refer to degradation and diluation rates due to cell growth. The term $g\frac{x_{4}}{x_{4}+K_{g}}$ describes the uptake of tryptophan for protein synthesis in the cell.

**Figure 8:**
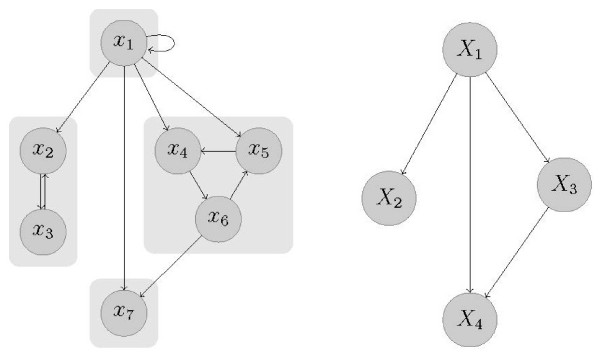
**Bifurcation diagrams of a model for tryptophan regulation in*Escherichia coli***. Bifurcation diagrams of the tryptophan regulation model (14) in *Escherichia coli* described in [11] with dilution rate *μ*as bifurcation parameter. The system shows two Hopf bifurcations at $\mu_{1}^{*}=0.02486$ and $\mu_{2}^{*}=0.1529$. Has been created with Additional file 5.

Analyzing this system with the parameter values given in [11] ($k_{1}=50\min^{-1},O_{t}=3.32\mathrm{nM},t_{1}=$$3.53\mathrm{\mu M},m_{1}=1.92,\gamma_{1}=0.5\min^{-1},\mu =0.01\mathrm{mi}n^{-1},$$k_{2}=15\mathrm{mi}n^{-1},t_{2}=0.04\mathrm{\mu M},m_{2}=1.72,\gamma_{2}=15\min^{-1},$$k_{3}=90\mathrm{mi}n^{-1},k_{4}=59\min^{-1},t_{3}=810\mathrm{\mu M},m_{3}=1.2,$*g*=25*μMK*_*g*_=0.2*μM*) using xppaut and choosing the dilution rate *μ*as bifurcation parameter, the system shows a Hopf bubble between $\mu_{1}^{*}=0.02486$ and $\mu_{2}^{*}=0.1529$ (Figure 8). The system has a unique fixed point that is unstable between these two values and shows sustained oscillations in this range. Outside the Hopf bubble the oscillations are damped and the fixed point is globally stable.

**Figure 9:**
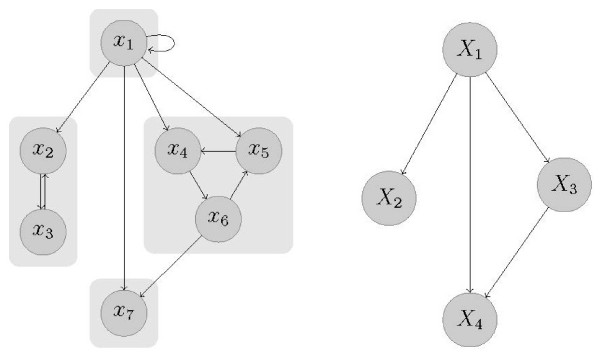
**I-graph of the tryptophan regulation network.** I-graph of the tryptophan regulation network (14).

The corresponding I-graph is shown in Figure 9. It is strongly connected.

The circuit set *C* and the minimal circuit covering vertex set $\tilde{V}$ are $C=\left\{\left\{x_{4}\},\{x_{2},x_{3},x_{4}\},\{x_{1},x_{2},x_{3},x_{4}\right\}\right\}$ and $\tilde{V}=\{x_{4}\}$. Since $\tilde{V}$ consists of a single element, this system is a LVG and only one circuit-closing step is necessary to calculate the set of fixed points. The circuit-characteristic can be calculated analytically here and is given by

(16) $$c(\kappa_{4})=r_{4}({\bar{x}}_{3}(\kappa_{4}),\kappa_{4})-\mu \kappa_{4},$$

where *r*_4_can iteratively be calculated via

(17) $$\begin{array}{lll} {\bar{x}}_{1}(\kappa_{4})\;\; & =\;\;\frac{r_{1}(\kappa_{4})}{\gamma_{1}+\mu }\; & \;\\ {\bar{x}}_{2}(\kappa_{4})\;\; & =\;\;\frac{r_{2}({\bar{x}}_{1}(\kappa_{4}),\kappa_{4})}{\gamma_{2}+\mu }\; & \;\\ {\bar{x}}_{3}(\kappa_{4})\;\; & =\;\;\frac{r_{3}({\bar{x}}_{2}(\kappa_{4}))}{\mu }.\; \end{array}$$

**Figure 10:**
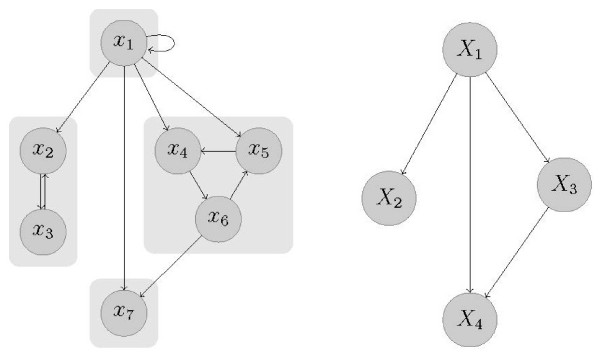
**Circuit-characteristics of the tryptophan regulation model.** Sets ${\bar{x}}_{i}(\kappa_{4})$ for *i*=1,2,3 and circuit-characteristic *c*(*κ*_4_) of the model of tryptophan regulation in *Escherichia coli* with the same parameter set as was used in [11] and dilution parameters *μ*=0.01 (*right column*), *μ*=0.1 (*center column*) and *μ*=0.2 (*left column*). Has been created with Additional file 6.

As can be seen in Figure 10, *c*(*κ*_4_) is strictly decreasing (*bottom row*), since all circuits in the graph are negative.

Furthermore, the fixed points of the system can be determined by the zeros of the characteristic, as depicted in the figure: For *μ*=0.01, for example, *c*(*κ*_4_) has a zero at $\kappa_{4}^{*}=31.8$, which corresponds to the fixed point coordinate ${\bar{x}}_{4}$. Inserting this value into ${\bar{x}}_{i}(\kappa_{4})$, *i*=1,2,3, we get the fixed point $\bar{x}(\mu =0.01)=(4.71,4.82\cdot 10^{-5},0.43,31.82)$, and likewise for the other dilution rates. The qualitative courses of *c*(*κ*_4_) and also for the fixed point sets ${\bar{x}}_{i}(\kappa_{4})$ do not differ for the three dilution rates. In particular, the slope of the characteristic is in all three cases negative at the zero. However, the bifurcation diagrams in Figure 3 indicate that the respective fixed points are stable for *μ*=0.01 and *μ*=0.2, but unstable for *μ*=0.1. Thus this is a further example that a negative slope of the characteristic at a zero does not imply stability of the respective fixed point.

## Conclusions

In this paper we have extended previous work on the analysis of fixed points for regulatory network models. Based on the circuit-breaking algorithm, which was introduced in [14] and which uses the topology of the interaction graph to construct a one-dimensional circuit-characteristic whose zeros correspond to the fixed points of the system, we further investigated this characteristic with respect to fixed points of the system and their stability. Here we demonstrated that the characteristic is in some aspects similar to a one-dimensional vector field and that the CBA is also useful to find fixed point bifurcations. Information about the stability of fixed points can partly be derived from the slopes at the respective zeros of the characteristic. We used our methods to analyze the fixed points of models for hematopoietic stem cell differentiation, tryptophan regulation in *Escherichia coli* and the repressilator in *Escherichia coli*. In particular, we have shown that a positive slope of the characteristic at a zero can imply instability, at least for certain graph topologies, which we call leading vertex graphs. These are characterized by leading vertices for all strongly connected components that are contained in all circuits. Although we have noticed that many network models belong to this model class, this restriction on the topology for sure limits the use of our approach. However, we believe that the implication can further be generalized to other network topologies, although a pure translation of the techniques that we are currently using is not possible. Thus a generalization is one topic for future work.

On the contrary, generally no conclusions about stability can be drawn from a negative slope, and the respective fixed point can either be stable or unstable. If it is unstable, we interpret this result as a kind of time-delay. This delay is due to the response time of the network to changes in the input *κ*_*i*_. It is not visible in the characteristic any more, where the effects of all feedback circuits have been summarized to a single effective one comprising only one component. This effect might be similar to a time-delay that destabilizes a stable fixed point in a one-dimensional vector field.

While this manuscript was in revision, we became aware of a recent paper [18] that seems to be closely related to our work in some aspects. In this paper, small phosphorylation motifs in signaling pathways are investigated subject to their ability to show bistable behavior. The authors follow the same idea of variable elimination to construct finally one-dimensional functions that contain information about the fixed points of the system and their stability. However, the techniques used therein are build on mass action kinetics and rational functions and explicitly use mass conservation relations. However, some of the mathematical ideas behind that seem to be related to our work, and a further comparison would be interesting.

Generally, the efficiency of the CBA and the analysis introduced here depends on the graph topology and the complexity to solve the implicit equations therein. Construction of the circuit-characteristic is particularly simple and efficient for graph topologies whose strongly connected components have minimal circuit-covering vertex set $\tilde{V}$ with only few elements, and thus our theory can be particularly helpful to analyze such networks.

In the future we will try to generalize results further, such that our approach is applicable to a broad range of regulatory network models. We will also further investigate the connection between the partial circuit-characteristics and the influence of the respective sets of circuits that are closed on the coordinates, number and stability of the system’s fixed points. We believe that our analysis can lead to the identification of circuit sets which are responsible for certain behaviors of the system that are connected to bifurcations of fixed points. Finally, we hope that we can contribute towards developing analysis methods that facilitate an understanding of the role of interrelated feedback circuits in regulatory network models for the system’s overall behavior.

## Methods

In this section we collect the mathematical technicalities that are needed to show the statements made in the Results and Discussion section of the manuscript.

### Proof of Proposition 1

This section shows the proof of Proposition 1. To avoid complex indexing, the relation is exemplarily shown on a fully connected 3-vertex network and a network with four vertices. These examples are non-trivial in the sense that the cardinality of the minimal covering vertex set, $\left|\tilde{V}\right|$, contains more than one element, such that calculation of the characteristic requires more than a single circuit-closing step. Thus the principles of these two examples can be generalized to other I-graphs.

#### 3-vertex model

##### Proof

We consider a regulatory network model with a fully-connected I-graph with three vertices:

(18) $$\begin{array}{cc} {\dot{x}}_{1} & =f_{1}(x_{1},x_{2},x_{3})=f_{1}(x)\\ {\dot{x}}_{2} & =f_{2}(x_{1},x_{2},x_{3})=f_{2}(x)\\ {\dot{x}}_{3} & =f_{3}(x_{1},x_{2},x_{3})=f_{3}(x), \end{array}$$

whose Jacobian matrix is given by

(19) $$\det\;J_{f}(x)\overset{\frac{\partial f_{i}(x)}{\partial x_{j}}:=f_{\mathrm{ij}}}{=}f_{11}f_{22}f_{33}+f_{12}f_{23}f_{31}+f_{13}f_{32}f_{21}$$

(20) $$-f_{11}f_{23}f_{32}-f_{13}f_{22}f_{31}-f_{12}f_{21}f_{33}.$$

**Figure 11:**
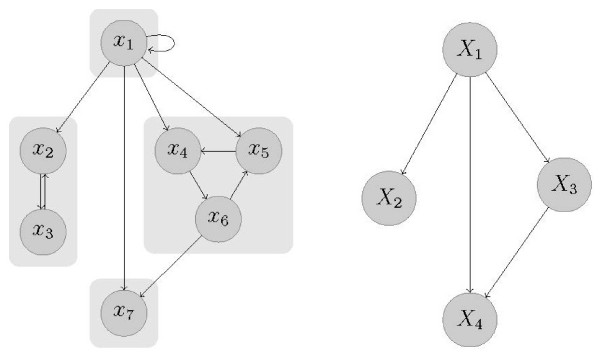
**Circuit-breaking algorithm for a regulatory network model with three vertices.** The circuit-breaking algorithm for a regulatory network model with three vertices and fully connected I-graph.

We now construct the circuit-characteristic *c*(*κ*_1_) using the CBA, whose steps are illustrated in Figure 11.

##### 

In order to calculate it’s derivative and show Proposition 1, we will repeatedly use the Implicit function theorem (IFT), which reads:

*Implicit function theorem*[19]: Let *U* be an open set in $R^{m}\times R^{n}$ and let $f:U\to R^{n}$ be a $C^{k}$ function with *k*≥1. Consider a point $(\bar{x},\bar{y})\in U$, where $\bar{x}\in R^{m}$ and $\bar{y}\in R^{n}$, with $f(\bar{x},\bar{y})=c$. If the *n*×*n* matrix $D_{y}f(\bar{x},\bar{y})$ of partial derivatives is invertible, then there are open sets $V_{m}\subset R^{m}$ and $V_{n}\subset R^{n}$ with $(\bar{x},\bar{y})\in V_{m}\times V_{n}\subset U$ and a unique *C*^*k*^ function *ψ*:*V*_*m*_→*V*_*n*_ such that *f*(*xψ*(*x*))=*c* for all *x*∈*V*_*m*_. Moreover, *f*(*xy*)≠*c* if (*xy*)∈*V*_*m*_×*V*_*n*_ and *y*≠*ψ*(*x*). The derivative of the function *ψ*is given by the formula

(21) $$\mathrm{D\psi}(x)=-{\left[D_{y}f(x,\psi (x))\right]}^{-1}D_{x}f(x,\psi (x)).$$

In the first step we break all circuits by fixing *x*_1_=*κ*_1_and *x*_2_=*κ*_2_(Figure 11*left*) and get the partial circuit-characteristic and the fixed point set

(22) $$f_{3}(\kappa_{1},\kappa_{2},x_{3})\overset{!}{=}0\;\Rightarrow \;{\bar{x}}_{3}(\kappa_{1},\kappa_{2})$$

with derivative given by

(23) $$\frac{d{\bar{x}}_{3}(\kappa_{1},\kappa_{2})}{d\kappa_{i}}\overset{\text{IFT}}{=}-f_{33}^{-1}f_{3i},\;i=1,2$$

Here we have used the IFT with *m*=2, *n*=1, $U=R^{3}$, $f:R^{3}\to R=f_{3}(\kappa_{1},\kappa_{2},x_{3})$, *c*=0, and $\psi (x)={\bar{x}}_{3}(\kappa_{1},\kappa_{2})$.

In the next step we release *v*_2_(Figure 11*center*) and get the partial circuit-characteristic and the fixed point set

(24) $$f_{2}(\kappa_{1},x_{2},x_{3}={\bar{x}}_{3}(\kappa_{1},x_{2}))\overset{!}{=}0\;\Rightarrow \;{\bar{x}}_{2}(\kappa_{1}),$$

with derivative

(25) $$\begin{array}{cc} \frac{d{\bar{x}}_{2}(\kappa_{1})}{d\kappa_{1}}\;\; & \overset{\text{IFT}}{=}\;\;-{\left(\frac{\partial f_{2}(\kappa_{1},x_{2},x_{3}={\bar{x}}_{3}(\kappa_{1},x_{2}))}{\partial x_{2}}\right)}^{-1}\\ \times \frac{\partial f_{2}(\kappa_{1},x_{2},x_{3}={\bar{x}}_{3}(\kappa_{1},x_{2}))}{\partial \kappa_{1}}\\ \;\; & =\;\;-{\left(\frac{\partial f_{2}(x)}{\partial x_{2}}+\frac{\partial f_{2}(x)}{\partial x_{3}}\frac{d{\bar{x}}_{3}}{dx_{2}}\right)}^{-1} \end{array}$$

(26) $$\times \left(\frac{\partial f_{2}(x)}{\partial x_{1}}+\frac{\partial f_{2}(x)}{\partial x_{3}}\frac{d{\bar{x}}_{3}}{d\kappa_{1}}\right)$$

(27) $$\;\overset{\text{eqn. 22}}{=}\;(\;-\underset{=:\beta^{-1}}{\underset{︸}{{\left(f_{22}\;-\;f_{23}f_{33}^{-1}f_{32}\right)}^{-1}}}\;\cdot (f_{21}-f{\;}_{23}f_{33}^{-1}f_{31})$$

Here we have used the IFT with *m*=1, *n*=1, $U=R^{2}$, $f:R^{2}\to R=f_{2}(\kappa_{1},x_{2},x_{3}(\kappa_{1},x_{2}))$, *c*=0, and $\psi (x)={\bar{x}}_{2}(\kappa_{1})$.

In the last step also vertex *v*_1_ is released (Figure 11*right*). The circuit-characteristic *c*(*x*_1_) reads:

(28) $$f_{1}(x_{1},{\bar{x}}_{2}(x_{1}),{\bar{x}}_{3}(x_{1},{\bar{x}}_{2}(x_{1})))\overset{!}{=}0\;\Rightarrow \;\{{\bar{x}}_{1}\},$$

and its derivative is given by

(29) $$\begin{array}{lll} \;{\frac{df_{1}(x)}{dx_{1}}|}_{\left(x_{1},F(x_{1})\right)}\;\; & =\;\;f_{11}+f_{12}\frac{d{\bar{x}}_{2}(x_{1})}{dx_{1}}+f_{13}\frac{d{\bar{x}}_{3}(x_{1},{\bar{x}}_{2}(x_{1}))}{dx_{1}}\; & \;\\ & =\;\;f_{11}-f_{12}\beta^{-1}(f_{21}-f_{23}f_{33}^{-1}f_{31})+f_{13}\; & \;\\ & \times \left(-f_{33}^{-1}f_{31}\;+\;f_{33}^{-1}f_{32}\beta^{-1}(f_{21}\;-\;f_{23}f_{33}^{-1}f_{31})\right)\; & \;\\ & =\;\;f_{11}-\beta^{-1}f_{12}f_{21}+\beta^{-1}f_{12}f_{23}f_{31}f_{33}^{-1}\; & \;\\ & -f_{13}f_{31}f_{33}^{-1}+\beta^{-1}f_{13}f_{32}f_{21}f_{33}^{-1}\; & \;\\ & -\beta^{-1}f_{13}f_{32}f_{23}f_{31}f_{33}^{-2}\; \end{array}$$

Multiplying this expression with $\det\;\overset{V\setminus \{v_{1}\}}{\underset{f}{J}}(x){\left|\right)}_{F(x_{1})}=f_{33}\cdot \beta$leads to

(30) $$\begin{array}{ll} \;\frac{df_{1}(x)}{dx_{1}}{|}_{\left(x_{1},F(x_{1})\right)}\cdot \det\;J_{f}^{V\setminus \{v_{1}\}}(x){|}_{F(x_{1})}{|}_{\left(x_{1},F(x_{1})\right)}\; & =\;\det\;J_{f}(x)\; \end{array}$$

□

#### 4-vertex model

##### Proof

**Figure 12:**
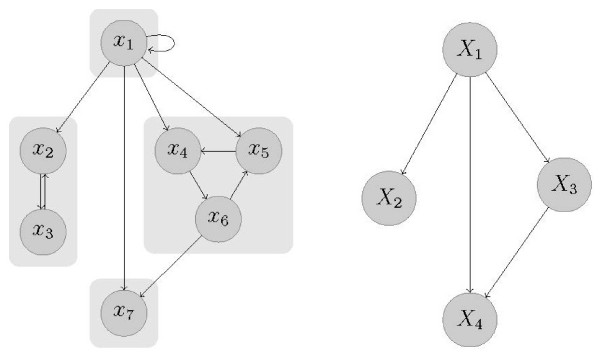
**Circuit-breaking algorithm for a regulatory network model with four vertices.** The circuit-breaking algorithm for a regulatory network model with four vertices.

Additionally, we outline the proof of proposition 1 for a non-trivial four-component network (Figure 12):

(31) $$\begin{array}{lll} {\dot{x}}_{1} & =\;f_{1}(x_{1},x_{3})=f_{1}(x)\; & \;\\ {\dot{x}}_{2} & =\;f_{2}(x_{1},x_{2},x_{4})=f_{2}(x)\; & \;\\ {\dot{x}}_{3} & =\;f_{3}(x_{1},x_{3},x_{4})=f_{3}(x)\; & \;\\ {\dot{x}}_{4} & =\;f_{4}(x_{2},x_{4})=f_{4}(x),\; & \; \end{array}$$

##### 

whose Jacobian matrix is given by

(32) $$\begin{array}{ll} \det\;J_{f}(x)= & f_{11}f_{22}f_{33}f_{44}+f_{13}f_{31}f_{24}f_{42}\;\\ & -f_{11}f_{33}f_{42}f_{24}-f_{13}f_{34}f_{42}f_{21}-f_{13}f_{31}f_{22}f_{44},\; \end{array}$$

where we used the same notation as before, i.e. $f_{\mathrm{ij}}:=\frac{\partial f_{i}(x)}{\partial x_{j}}$.

Again we construct the circuit-characteristic *c*(*κ*_1_) using the CBA and the IFT for it’s derivatives. First we break all circuits by fixing *x*_1_=*κ*_1_ and *x*_2_=*κ*_2_(Figure 12*left*) and calculating the fixed point coordinates of the remaining vertices:

(33) $$f_{4}(\kappa_{2},x_{4})\overset{!}{=}0\;\Rightarrow \;{\bar{x}}_{4}(\kappa_{2}),$$

with derivatives

(34) $$\frac{d{\bar{x}}_{4}(\kappa_{2})}{d\kappa_{1}}=0\;\text{and}\;\frac{d{\bar{x}}_{4}(\kappa_{2})}{d\kappa_{2}}\overset{\text{IFT}}{=}-f_{44}^{-1}f_{42},$$

and

(35) $$f_{3}(\kappa_{1},x_{3},{\bar{x}}_{4}(\kappa_{2}))\overset{!}{=}0\;\Rightarrow \;{\bar{x}}_{3}(\kappa_{1},{\bar{x}}_{4}(\kappa_{2})),$$

with derivatives

(36) $$\begin{array}{cc} \frac{d{\bar{x}}_{3}(\kappa_{1},{\bar{x}}_{4}(\kappa_{2}))}{d\kappa_{1}} & \overset{\text{IFT}}{=}-{\left(\frac{\partial f_{3}(\kappa_{1},\kappa_{3},{\bar{x}}_{4}(\kappa_{2}))}{\partial x_{3}}\right)}^{-1}\\ \;\;\times \frac{\partial f_{3}(\kappa_{1},x_{3},{\bar{x}}_{4}(\kappa_{2}))}{\partial \kappa_{1}} \end{array}$$

and

(37) $$\begin{array}{lll} \frac{d{\bar{x}}_{3}(\kappa_{1},{\bar{x}}_{4}(\kappa_{2}))}{d\kappa_{2}}\;\; & \overset{\text{IFT}}{=}\;\;-{\left(\frac{\partial f_{3}(\kappa_{1},x_{3},{\bar{x}}_{4}(\kappa_{2}))}{\partial x_{3}}\right)}^{-1}\; & \;\\ & \;\;\;\;\times \frac{\partial f_{3}(\kappa_{1},x_{3},{\bar{x}}_{4}(\kappa_{2}))}{\partial \kappa_{2}}\;\\ \;\; & =\;\;-f_{33}^{-1}\left(f_{34}\frac{d{\bar{x}}_{4}(\kappa_{2})}{d\kappa_{2}}\right)\;\\ \;\; & \overset{\text{eqn.}\;(\text{31})}{=}\;\;f_{33}^{-1}f_{34}f_{42}f_{44}^{-1}\; \end{array}$$

In the next step we release *v*_2_(Figure 12*center*) and get the partial circuit-characteristic and the fixed point set

(38) $$f_{2}(\kappa_{1},x_{2},{\bar{x}}_{4}(x_{2}))\overset{!}{=}0\;\Rightarrow \;{\bar{x}}_{2}(\kappa_{1}),$$

whose derivative is given by

(39) $$\begin{array}{lll} \frac{d{\bar{x}}_{2}(\kappa_{1})}{d\kappa_{1}}\;\; & \overset{\text{IFT}}{=}\;\;-{\left(\frac{\partial f_{2}(\kappa_{1},x_{2},{\bar{x}}_{4}(x_{2}))}{\partial \kappa_{1}}\right)}^{-1}\; & \;\\ & \;\;\;\;\times \frac{\partial f_{2}(\kappa_{1},x_{2},{\bar{x}}_{4}(x_{2}))}{\partial \kappa_{1}}\;\\ \;\; & =\;\;-{\left(f_{22}+f_{24}\frac{d{\bar{x}}_{4}(x_{2})}{dx_{2}}\right)}^{-1}\cdot f_{21}\;\\ \;\; & \overset{\text{eqn. (31)}}{=}\;\;-{\left(f_{22}-f_{24}f_{42}f_{44}^{-1}\right)}^{-1}\cdot f_{21}\; \end{array}$$

Thus we have expressed the fixed point coordinates of *x*_3_ and *x*_4_ in terms of *κ*_1_, ${\bar{x}}_{3}(\kappa_{1})={\bar{x}}_{3}(\kappa_{1},{\bar{x}}_{4}({\bar{x}}_{2}(\kappa_{1})))$ and ${\bar{x}}_{4}(\kappa_{1})={\bar{x}}_{4}({\bar{x}}_{2}(\kappa_{1}))$. Finally we release *v*_1_. The circuit-characteristic *c*_1_(*x*_1_) reads:

(40) $$f_{1}(x_{1},{\bar{x}}_{3}(x_{1},{\bar{x}}_{4}({\bar{x}}_{2}(x_{1}))))\overset{!}{=}0\;\Rightarrow \;\{{\bar{x}}_{1}\},$$

and its derivative is given by

(41) $$\begin{array}{lll} \;\frac{df_{1}(x_{1},{\bar{x}}_{3}(x_{1},{\bar{x}}_{4}({\bar{x}}_{2}(x_{1}))))}{dx_{1}} & =f_{11}+f_{13}\frac{d{\bar{x}}_{3}(x_{1},{\bar{x}}_{4}({\bar{x}}_{2}(x_{1})))}{dx_{1}}\;\\ & =f_{11}+f_{13}\left(\frac{\partial {\bar{x}}_{3}(x_{1},x_{4})}{\partial x_{1}}\right)\; & \;\\ & \left(+\frac{\partial {\bar{x}}_{3}(x_{1},x_{4})}{\partial x_{4}}\frac{\partial {\bar{x}}_{4}(x_{2})}{\partial x_{2}}\right)\; & \;\\ & \times \left(\frac{d{\bar{x}}_{2}(x_{1})}{dx_{1}}\right).\; \end{array}$$

Using again the IFT to eliminate derivatives of fixed point coordinates, i.e.

(42) $$\frac{d{\bar{x}}_{3}}{dx_{1}}=-f_{33}^{-1}f_{31}$$

(43) $$\frac{d{\bar{x}}_{3}}{dx_{4}}=-f_{33}^{-1}f_{34}$$

(44) $$\frac{d{\bar{x}}_{4}}{dx_{2}}=-f_{44}^{-1}f_{42}$$

(45) $$\frac{d{\bar{x}}_{2}}{dx_{1}}=-{\left(f_{22}-f_{24}f_{42}f_{44}^{-1}\right)}^{-1}f_{21},$$

the derivative of the characteristic becomes

(46) $$\frac{df_{1}(x_{1},{\bar{x}}_{3}(x_{1},{\bar{x}}_{4}({\bar{x}}_{2}(x_{1}))))}{dx_{1}}$$

(47) $$=\frac{df_{1}(x_{1})}{dx_{1}}{\left|\right)}_{\left(x_{1},F(x_{1})\right)}$$

(48) $$\begin{array}{c} =f_{11}+f_{13}\left(-f_{33}^{-1}f_{31}+f_{33}^{-1}f_{34}(-f_{44}^{-1}f_{42})\right)\\ \;\left(\times {\left(f_{22}-f_{24}f_{42}f_{44}^{-1}\right)}^{-1}\cdot f_{21}\right). \end{array}$$

Setting $f_{22}-{f_{24}f_{42}f_{44}^{-1})}^{-1}=:\beta^{-1}={\left(f_{33}f_{44}\right)}^{-1}\det\;\overset{V\setminus \{x_{1}\}}{\underset{f}{J}}(x){|}_{F(x_{1})}$, we can see that equation (49) equals $\det\;J_{f}(x){|}_{\left(x_{1},F(x_{1})\right)}$ when multiplied with *βf*_33_*f*_44_. This can easily be seen by multiplying (49) out and rearranging the order of the summands. □

### Unstable fixed points in LVGs

Since for LVGs the subnetwork spanned by the set *V*∖{*v*_1_} does by definition not contain any circuits, we get a simple expression for $\det\;\overset{V\setminus \{v_{1}\}}{\underset{f}{J}}(x){\left|\right)}_{F(x_{1})}$, namely:

(49) $$\det\;\overset{V\setminus \{v_{1}\}}{\underset{f}{J}}(x){\left|\right)}_{F(x_{1})}=\overset{n}{\underset{i=2}{\prod }}\frac{\partial f_{i}(x)}{\partial x_{i}}{\left|\right)}_{\left(x_{1},F(x_{1})\right)},$$

with $\frac{\partial f_{i}(x)}{\partial x_{i}}{\left|\right)}_{\left(x_{1},F(x_{1})\right)}<0$ for all *i*. Thus, the sign of this expression is given by

(50) $$\sigma \left(\det\;J_{f}^{V\setminus \{v_{1}\}}(x){\left|\right)}_{\left(x_{1},F(x_{1})\right)}\right)=\left\{\begin{array}{ll} - & n\;\text{even}\\ + & n\;\text{odd} \end{array}\right)$$

Now we assume that $\bar{x}=({\bar{x}}_{1},F({\bar{x}}_{1}))$ is stable, and hence *R*(*λ*)<0 for all eigenvalues *λ*of $J_{f}(\bar{x})$. It follows that

(51) $$\sigma \left(\det\;\;J_{f}(\bar{x})=\overset{n}{\underset{i=1}{\prod }}\lambda_{i}\right)=\left\{\begin{array}{ll} + & n\;\text{even}\\ - & n\;\text{odd}\; \end{array}\right)$$

In any case, $\det\;J_{f}(\bar{x})$ and $\det\;\overset{V\setminus \{v_{1}\}}{\underset{f}{J}}(\bar{x})$ have different signs, a contradiction to $\frac{\mathrm{dc}(\kappa_{1})}{d\kappa_{1}}{\left|\right)}_{\kappa_{1}^{*}}>0$, which completes the proof.

### Verification of Proposition 1 for the repressilator model

Let us verify Proposition 1 for the repressilator model. We can identify

(52) $$\begin{array}{cc} \;\det\;J_{f}(\bar{x}) & =\beta^{3}-\beta^{3}\\ \;\times \left(\frac{\mathrm{\partial r}(p_{3})}{\partial p_{3}}{\left|\right)}_{{\bar{p}}_{3}=r(r(\kappa_{1}^{*}))}\frac{\mathrm{\partial r}(p_{1})}{\partial p_{1}}{\left|\right)}_{{\bar{p}}_{1}=\kappa_{1}^{*}}\frac{\mathrm{\partial r}(p_{2})}{\partial p_{2}}{\left|\right)}_{{\bar{p}}_{2}=r(\kappa_{1}^{*})}\right) \end{array}$$

and $\det\;\overset{V\setminus \{m_{1}\}}{\underset{f}{J}}(\bar{x})=-\beta^{3}$, such that both $\det\;J_{f}(\bar{x})$ and $\det\;\overset{V\setminus \{m_{1}\}}{\underset{f}{J}}(\bar{x})$ depend on *β*, but *c*(*κ*_1_) does not, since *β* is canceled out. The derivative of *c*(*κ*_1_) (equation (13)) with respect to *κ*_1_reads

(53) $$\;\frac{\mathrm{dc}(\kappa_{1})}{d\kappa_{1}}{\left|\right)}_{\kappa_{1}^{*}}=\frac{\mathrm{\partial c}}{\partial \kappa_{1}}+\frac{\mathrm{\partial r}(r(r(\kappa_{1})))}{\mathrm{\partial r}(r(\kappa_{1}))}\frac{\mathrm{\partial r}(r(\kappa_{1}))}{\mathrm{\partial r}(\kappa_{1})}\frac{\mathrm{dr}(\kappa_{1})}{d\kappa_{1}}{\left|\right)}_{\kappa_{1}^{*}}$$

(54) $$=-1+r^{\prime \prime }(x){\left|\right)}_{x=r(r(\kappa_{1}^{*}))}r^{\prime \prime }(x){\left|\right)}_{x=r(\kappa_{1}^{*})}r^{\prime \prime }(x){\left|\right)}_{x=\kappa_{1}^{*}},$$

which equals $\det\;J_{f}(\bar{x})\cdot \overset{-1}{\det}J_{f}^{V\setminus \{x_{1}\}}(\bar{x})$.

## Endnotes

^1^This plot was created with the program xppaut and Additional file 7 in the Supplement (tryptophanmodel.ode)

^2^This plot was generated with the program gnuplot and Additional file 8 (tryptophanmodel-c.gp) in the Supplement.

## Competing interests

The authors declare that they have no competing interests.

## Author’s contributions

NR designed research and carried out calculations on the examples.

## Additional files

## Supplementary Material

Addtional file 1 **Stemcellmodel.** This file was used to create the bifurcation diagrams of the hematopoietic stem cell model using the program xppaut (Figure 3). Click here for file

Addtional file 2 **Stemcellmodel-02.** The file stemcellmodel-02.gp was used to create the circuit-characteristic *c*(*κ*) and fixed point sets *F*(*κ*) of the hematopoietic stem cell model (Figure 5) with bifurcation parameter *μ*=0.2 using the program gnuplot. Click here for file

Addtional file 3 **Stemcellmodel-bif.** The file stemcellmodel-bif.gp was used to create the circuit-characteristic *c*(*κ*) and fixed point sets *F*(*κ*) of the hematopoietic stem cell model (Figure 5) with bifurcation parameter *μ*=*μ*^∗^using the program gnuplot. Click here for file

Addtional file 4 **Stemcellmodel-05.** The file stemcellmodel-05.gp was used to create the circuit-characteristic *c*(*κ*) and fixed point sets *F*(*κ*) of the hematopoietic stem cell model (Figure 5) with bifurcation parameter *μ*=0.5 using the program gnuplot. Click here for file

Addtional file 5 **Newton-2d.** This python script was used to calculate the eigenvalues of the Jacobian matrix $J_{f}(\bar{x})$ of the repressilator model at it’s fixed point using a Newton gradient search with random starting points. These eigenvalues were written into the file ‘eigenvalues.txt’. Click here for file

Addtional file 6 **Eigenvalues.** This file contains the eigenvalues of the Jacobian matrix $J_{f}(\bar{x})$ of the repressilator model at it’s fixed point and was created by running the program newton-2d.py. It was used to create Figure 7. Click here for file

Addtional file 7 **Tryptophanmodel.** This file was used to create the bifurcation diagrams of the tryptophan regulation model using the program xppaut (Figure 8). Click here for file

Addtional file 8 **Tryptophanmodel-c.** This file was used to create the circuit-characteristic *c*(*κ*) and fixed point sets *F*(*κ*) of the tryptophan regulation model (Figure 10) using the program gnuplot. Click here for file
